# Tyrant’s End: Did Joseph Stalin Die From Warfarin Poisoning?

**DOI:** 10.7759/cureus.36265

**Published:** 2023-03-16

**Authors:** Matthew D Turner

**Affiliations:** 1 Hospital-Based Medicine, Madigan Army Medical Center, Lakewood, USA

**Keywords:** acute poisoning, poisoning, mca stroke, medical history, stroke, warfarin toxicity, joseph stalin

## Abstract

One of the most brutal dictators in humanity’s history, Joseph Stalin forged the Soviet Union into a massive superpower, crushing the lives of millions of his own citizens along the way. His sudden death in March 1953 from a stroke took the world by surprise and led to a frantic power struggle within the ranks of the Soviet government. In recent years, researchers have proposed that Stalin’s stroke was not natural and was potentially caused by one of his lieutenants poisoning him with warfarin or a similar anticoagulant. After examining the evidence, this article concludes that both Stalin’s disease course and the properties of warfarin make it highly unlikely that he was deliberately assassinated.

## Introduction and background

In the early days of March 1953, as the rest of the world waited with bated breath for news out of the Soviet Union, an American ambassador sarcastically wrote: “Uncle Joe is sick in bed - Rush in blood up in his head - If he can’t walk and he can’t talk - By whom now are the Communists led?” [1]. The mood was far more serious in Moscow, for Joseph Stalin, absolute dictator of the Union of Soviet Socialist Republics (USSR) for nearly 30 years [2], was dying. As his physicians desperately tried to treat his symptoms, his underlings jockeyed for position in the subsequent power vacuum. By March 5th, Stalin’s last battle was over, and the dictator finally lay dead [2]. The USSR officially declared that his cause of death was a hemorrhagic stroke [3]. However, recent evidence has come to light that throws this into doubt, with some researchers maintaining the possibility that Stalin was actually poisoned, likely by warfarin or a similar drug with anticoagulant properties [4-6]. This article seeks to examine the available evidence to determine if Stalin’s stroke was naturally occurring or artificially induced.

## Review

Stalin’s health

Before a thorough discussion of Stalin’s death can occur, his life must also be addressed. The man that the world would know as Joseph Stalin, the “organizer and director of one of the most powerful and merciless terror machines known to history” [2] was born Ioseb Jughashvili in the small town of Gori, modern-day Georgia, on the outskirts of the Russian Empire. Although Soviet propaganda claimed he was born on 21 December 1879, he was actually born on or about 6 December 1878. To this day, the reason for this date change is unknown [2], but it does illustrate the bizarre mix of mythologizing and outright fabrication that went into the creation of his legendary cult of personality decades later [2]. From birth, he was afflicted by a number of health issues. The only one of three children to survive to adulthood, he was born with two fused toes on his left foot, and in his early years had an early bout of smallpox that left his face permanently pockmarked [2], suffered from measles, scarlet fever, was involved in three separate traffic accidents, and experienced a mysterious atrophying of his left arm [7]. To this day, it is still unclear what happened to Stalin’s limb, which was shorter than his right arm by approximately 5.5 cm and so useless that it was a “practical disability” [7]. His daughter Svetlana thought it may have been due to a birth injury [7]; some researchers have agreed that Stalin may have experienced Erb’s Palsy [8]. Still others have proposed that it was the result of a childhood injury [2]. Whatever the original cause, it would plague him for the rest of his life.

The following decades of his life were even harder on his health. The now-revolutionary, exiled to the harsh and austere environment of Siberia, experienced a bout of typhus in 1909, followed by pulmonary tuberculosis in 1917. During the Russian Civil War, he underwent an urgent appendectomy in 1921, with complications so significant that his surgeon thought his death was almost certain [2]. Even after such a brush with death, he had to be practically strong-armed by the Politburo into taking an extended vacation for his own health [2]. He developed chronic headaches the next year [8], along with chronic digestive issues that caused him recurrent abdominal pain with significant diarrhea and flatulence [2]. By the 1930s, Stalin experienced occasional angina in addition to hypertension and recurrent joint and muscle pains [2].

By nearly all accounts, Stalin entered World War II - dubbed “The Great Patriotic War” by the USSR [2] at the height of his abilities, with “disciplined and formidable mental powers” [7]. Although Stalin’s failure to anticipate Operation Barbarossa in 1941 was a grievous mistake that nearly cost him everything, it does not appear to have a medical etiology. As he received news of German forces mobilizing on the border, he refused to allow Soviet forces to prepare in order to avoid a provocation - a strategy that he had successfully used in 1930 and 1939 border clashes with Poland and Japan, respectively. In light of his previous experience, Stalin’s error can be attributed to a costly miscalculation and not to medical incompetence [9]. Regardless, the war itself was a “grueling experience” for Stalin, who served in five leadership roles during the conflict. In October 1945, he went on his first vacation in nearly a decade - rumors abounded that this may have been due to a minor myocardial infarction or sheer exhaustion [10]. There is a possibility that he experienced a second heart attack in 1948, along with minor strokes in 1945 and 1947 [8]. As his health deteriorated, so did his mental state, as the dictator experienced dizziness, worsening memory, increased aggressiveness, and increased paranoia [2]. Even to his contemporaries, the dictator looked visibly unhealthy, appearing as a “tired old man… (who) talked with difficulty” [8]. A visiting Yugoslavian communist in 1948 described Stalin’s unnaturally pale face with “ruddy cheeks,” a distinctive appearance so common amidst the higher-ups of the Party that it was dubbed the “Kremlin complexion” [8]. Even his famed mustache was thinning [11].

By 1952, Stalin’s health, worsened by advancing atherosclerosis [2] had become so poor that his personal physician, cardiologist Dr. Vladimir Vinogradov, advised him on 19 January to resign from ruling for his own health [1]. Stalin angrily rebuffed him and, aside from a brief visit to an ENT specialist in April of that year for a severe cold, does not appear from the records available to us to have ever seen a doctor again [1,2]. In November, Vinogradov was arrested by the Ministry of State Security (MGB) and tortured as part of the lead-up to the Doctors’ Plot [1].

Before discussing the Doctor’s Plot, it is necessary to establish Stalin’s history with healthcare professionals. In one of the most important moments of the 20th century, the “Big Three” leaders of the Allies met at the 1945 Yalta Conference to lay out the course of postwar Europe. Interestingly, all three of the major leaders - Winston Churchill, Franklin Delano Roosevelt, and Joseph Stalin - suffered from hypertension, and all three would die of strokes [12]. Of the three, Stalin is the only one who had a history of having his physicians killed. Eighteen years before the Yalta Conference, world-renowned neurologist Dr. Vladimir Bechterev had an interview with Stalin. Later that day, he privately told his assistant that the dictator was “paranoiac”. By the next day, the neurologist had been poisoned [13]. Another physician named Dr. Pletnev diagnosed Stalin with “megalomania and a persecution complex” in 1937, just before his arrest. He was executed by the firing squad in 1941 [8]. Vinogradov’s 1952 arrest followed this pattern, for Stalin had “developed a pathological fear of medical professionals” [1]. While this may have been due to Stalin’s ever-expanding paranoia, his personal history may have played a factor here. When Vladimir Lenin suffered a series of strokes at the end of his life, Stalin personally took over his care and skillfully leveraged Lenin’s incapacity to secure the reins of the USSR’s government for himself [14]. As someone who had personally used healthcare to seize power, he may have feared that someone would do the same to him.

In his later years, the only physician that Stalin ever showed a measure of respect for was the famous Dr. Lina Shtern, even going so far as to reduce a death sentence for her for being in the Jewish Anti-Fascist Committee (JAC) to a prison sentence. Rumors abounded that he spared the famous biochemist and winner of the 1943 Stalin Prize because of her research regarding longevity [1].

The government crackdown on the JAC and Vinogradov’s 1952 arrest were direct precursors to the infamous Doctor’s Plot. Less than two months after his arrest, the January 13th, 1953 edition of *Pravda *officially announced the discovery of a “terrorist group of doctors who had made it their aim to cut short the lives of active public figures of the Soviet Union” [15]. Included in the list of nine “murderer-doctors” rounded up by the MGB were Dr. Shimeliovich, chief doctor of Moscow’s Botkin Hospital, one of the most prestigious in the USSR [15], and Dr. Yegorov, who had been chief of the Kremlin’s medical directorate and thus had likely treated most of the Politburo at one time or another, including Stalin himself [16]. Though the impetus for the Doctors’ Plot may have been due to Vinogradov’s January 1952 warning to Stalin of his own mortality, as some sources suspect [7], the majority of the physicians named in the Pravda article were Jewish, a fact not lost on the Soviet population [15]. From January to March, the Soviet government enacted a vast program of state-sanctioned anti-Semitism against the 2,000,000 Jews that called the USSR home. Over a million copies of one pamphlet, menacingly titled “Why Jews Must Be Resettled from the Industrial Regions of the Country” were prepared, as well as the construction of vast camps in Siberia [13]. The terror swiftly percolated down throughout all society, as mothers refused to give medicines to their children, thinking it was “poison,” and in many hospitals “patients regarded the doctors as insidious scoundrels and refused to take any medicines” [1]. The true planned extent of the Doctors’ Plot will likely never be known, for Stalin, its chief architect, died before it could be completed [2]. His successors quickly distanced themselves from the campaign, and an April 4th *Pravda *article even admitted that it had been fabricated. The MGB soon released seven of the arrested doctors - the remaining two had already died in captivity [13].

As some of the most talented physicians in the USSR were being tortured to confess their collusion with “foreign intelligence services” [15], Stalin continued to display the “ruthless insistence on total control at all times” that had defined him since childhood in nearly every aspect of his life, from political domination [7] to planning in painstaking detail the exact minutiae of his private dacha outside Moscow that he would eventually die in [2]. It is curious that he never once applied this same attitude towards his health. Throughout his life, Stalin had a notable “penchant for immobility,” barely engaging in any sort of exercise at all [2]; even going for a walk in his later years was out of the question [8]. Although his exile into Siberia by the Tsarist government as a young man forced him to learn hunting out of necessity, he abandoned the activity as soon as he was able [2]. He consistently displayed “no interest in sports or any physical activity more strenuous than billiards” [11], chain-smoked until he reluctantly quit in 1952 [7], ate an excess of rich foods “with an emphasis on meat dishes,” and overworked [2]. Stalin’s insomniac habit of total dedication to work was expected of all Party members, so much so that a 1947 government report found many of the upper echelons of the USSR’s administration suffered from “diseases of the heart and the circulatory and nervous systems sufficiently serious to impact their ability to work… one cause of (this) is stressful work not only during the day, but also during the night, and often even on holidays” [2]. Alcohol was the one substance that he practiced in moderation throughout his life, preferring to sip on light Georgian wine while he forced his guests to drink to excess [2].

There is no archival evidence that Stalin was under the care of any physician since January 1952, aside from the brief visit to an ENT specialist in April that has already been discussed [2]. In the last months of his life, his only form of healthcare was a few drops of iodine poured into a glass of water to treat his hypertension [2]. One of his primary medical advisors was a bodyguard who had previously received some training as a veterinarian [8]. Even his daughter Svetlana was surprised at the “quack” treatments her father was taking when she visited him [2].

The death of Stalin

It is in this environment of malaise, paranoia, and ill health that Joseph Stalin, Premier of the Soviet Union, found himself on the night of February 28th, 1953. At his private dacha in the suburbs of Moscow, he had a private gathering of the* Piaterka* - the “Five,” four of his most powerful underlings and himself, known to the others simply as “the Boss”. Included in the Five that night were Lavrentiy Beria, the ambitious and sadistic chief of Stalin’s secret police, Georgy Malenkov, Deputy General Secretary of the USSR, Nikita Khrushchev, future Premier of the Soviet Union, and Nikolai Bulganin, the Minister of Defense [2]. As this informal group, one that wielded absolute authority over the USSR [2], met for dinner, one of the topics that came up was the continued interrogation of Vinogradov. When pressed by Stalin, Beria admitted that the MGB had discovered that the cardiologist had told at least one other physician of Stalin’s “hypertonic episodes.” Stalin was not pleased to hear this, and demanded that Beria put further pressure on Semyon Ignatiev of the MGB to get “full confessions” before he “reduce(d) his height by a head.” Beria quickly agreed, and the conversation turned to other matters [6].

Khrushchev would later claim in his memoirs that Stalin was otherwise in a good mood that night, “tipsy” from consuming more wine than usual [8], although this has been contradicted by a former bodyguard who was also present, and described years later that Stalin was in a “bad humor” and consumed only light drinks [6]. Whatever the case, the gathering continued until approximately 4:00 AM on 1 March [3]. Stalin retired to his quarters, and for much of the rest of the day, none of the guards or servants heard anything out of the ordinary [3]. This was not particularly unusual for Stalin, who often went to bed in the early hours of the morning and typically woke up around 11:00 AM [2]. As noon came and went, his guards began to grow more and more concerned, but did not dare violate his strict instructions that no one was to disturb him “under any circumstances” [3]. One of the sentries reported at 6:30 PM that he saw Stalin’s dining room light come on, perhaps indicating that the dictator was busy with work [6], but the guards’ “sheer panic” continued to grow as the hours went on [6]. It wasn’t until 11:00 PM on 1 March that his housekeeper dared to enter his room. She found the dictator unconscious on the floor, dressed in a shirt and his pajama trousers. He was “unconscious, breathing heavily, incontinent, and unresponsive” [3], though by some accounts he was barely aware enough to attempt to speak, but only made a “strange buzzing sound” [1]. It had been nearly 18 hours since his last known normal state - although one of the guards would later claim that Stalin had a pocket-watch beside him that read 6:30 PM, implying that the stroke occurred at approximately the same time that the dining room light was seen [6]. Even with this updated timeline, it still implies that Stalin had lain unconscious on the floor for approximately 4.5 hours.

As some of the guards moved Stalin to a large sofa in the guest room, one of the others frantically called the head of the MGB, Semyon Ignatiev. Frightened, he ordered them to call Malenkov and Beria instead [1]. Beria immediately ordered them to not contact anyone else, and arrived at the dacha with Malenkov some four hours later, at 3:00 AM on 2 March. They performed a perfunctory exam of the seemingly-asleep Stalin, and Beria angrily exclaimed, “What are you panicking for? The Boss is sound asleep. Let’s go, Malenkov!” [6]. Ignoring the guards’ protests, they attributed Stalin’s state to alcohol from the night before and left [3,6]. Rubenstein theorizes that both men, far from foolishly dismissing the guards’ concerns, could not have missed the fact that a “serious medical incident” had occurred - although he was covered by a blanket, Stalin’s clothes had not been changed, and all those present could “smell how he soiled himself”. Perhaps Malenkov and Beria decided that it was “best for all concerned… to let him die” [1].

Regardless, the growing terror of the dacha came to the attention of Khrushchev, who arrived at the facility at approximately 7:30 AM that same day. Shocked by what he saw, he quickly summoned the ruling group and a group of doctors that arrived an hour later [10]. After 9.5 hours Stalin had been found, and 5.5 hours after he had been examined by Beria and Malenkov, medical aid finally arrived [10].

While Stalin’s own personal physician was being tortured in the depths of a prison cell, a hastily-assembled team of doctors examined the dictator [17]. They were so frightened by the experience that “their hands were too shaky” for a proper examination - the dentist shook with so much fear that he accidentally dropped Stalin’s dentures on the floor after removing them [6]. After examining Stalin, their prognosis was grim. He had a blood pressure of 190/110, was soaked in urine [3], had hemiplegia of his right arm and leg, and had a right-sided Babinski reflex [12]. They diagnosed him with a hemorrhage of the left middle cerebral artery (MCA) [17].

Over the next several days, the physicians would attempt a number of treatments, including “absolute quiet; the application of leeches to his ears; a cold compress on his head; an enema of milk of magnesia, and the removal of his false teeth” [1]. Mid-interrogation, three of the arrested physicians from the Doctors’ Plot were frantically consulted for medical advice on the treatment of stroke victims; at one point MGB agents suddenly asked an imprisoned pathologist what “Cheyne-Stokes respiration” was and if he could recommend any physicians to treat a “very important stroke patient.” All of the doctors that he recommended had already been arrested [1]. None of the treatments worked. Stalin’s blood pressure rose to 210/120 in spite of a second application of leeches, and his breathing became ragged and uneven as his pulse simultaneously became tachycardic and irregular. He was also given injections of magnesium sulfate in an attempt to lower his blood pressure as well as “hourly injections of camphora, strophantin, caffeine, glucose,” and supplemental oxygen [3].

As the dacha became crowded with a “swirl of anxious chaos,” the upper echelons of the Party gathered to witness the death of their leader - and to plan for the subsequent power vacuum that followed. Beria in particular seemed to be overjoyed, although Khrushchev noted that on the few instances where Stalin briefly seemed to regain some semblance of consciousness, Beria “threw himself on his knees, seized Stalin’s hand, and started kissing it” [1]. Most damningly of all, Beria was heard to brag to others that “I took him out” [3].

By 3 March, the doctors announced that “Death is inevitable” [1]. The Soviet government released an official announcement on Stalin’s health the next morning, admitting that “the grave illness of Comrade Stalin will involve his more or less prolonged non-participation in leading activity” [1]. As the world absorbed the shocking news, it became more and more clear that the dictator was on his deathbed [1]. For the few remaining hours of Stalin’s life, the government released continuing medical bulletins that “carefully outlined the nature of Stalin’s illness and meticulously described the measures being taken by the doctors who were treating him.” A top secret CIA report on Stalin’s death, declassified in June 2007, concluded that the USSR government was being extraordinarily careful to avoid any implications that Stalin’s care was being sabotaged by any “doctor wreckers” like in the alleged Doctors’ Plot [18].

On 5 March, Stalin’s condition worsened. He vomited blood on two occasions [7], significantly reducing both his blood pressure and his pulse [1]. This sudden development took the physicians by surprise and aroused the suspicion of Bulganin, who was present at the time. He demanded an explanation, and was given the theory that Stalin had developed small hemorrhages in his stomach lining. Bulganin was not convinced, and asked mockingly, “Is it possible? Perhaps Stalin has stomach cancer?” [1].

By then, there was nothing that could be done. Svetlana described her father’s “death agony” as “horrible,” stating “he literally choked to death as we watched.” With his last breath, Stalin “suddenly opened his eyes” and cast a “terrible glance, insane or perhaps angry and full of the fear of death” over the entire room, raising his left hand in a strange gesture as if “bringing down a curse… the gesture was incomprehensible and full of menace, and no one could say to whom or at what it might be directed” [1]. Seconds later, he expired at 9:50 PM on 5 March.

But it was not quite the end. To Khrushchev’s horror, one of the aids rushed to the bed and started giving Stalin artificial respiration, “massaging him to get him breathing again” [1]. This comes as no surprise as “doctors in totalitarian states are terrified of eminent dead patients” [19]. The man was quickly stopped, and the room fell into a profound silence, gazing at the “pitiable, poor corpse” that lay before them [1].

The subsequent autopsy was published in the 7 March edition of *Pravda* and revealed a large hemorrhage in the subcortical areas of the left cerebral hemisphere, as well as significant hypertrophy of Stalin’s left ventricle, numerous hemorrhages in the myocardium, stomach, and intestines, and significant atherosclerotic changes throughout his blood vessels. Pravda concluded that “all treatment attempts could not have led to a favorable outcome and prevent a fatal end” [3]. The description of Stalin’s brain suggests that he possibly had a number of lacunar infarcts, consistent with his history of reported strokes and secondary to his atherosclerosis [8]. Much like Lenin before him, Stalin’s brain was removed and sent to the Moscow Brain Institute for study. However, it remains unclear if the organ is still secure in a restricted section of the institute (now named the Brain Research Department of the Research Center of Neurology), if it is stored in another place entirely, or if it even still exists [3]. The autopsy report in *Pravda* was brief, and did not describe the neuropathology of Stalin’s brain in significant detail [3]. Nor does the final report made to the Central Committee of the USSR, which officially ruled that the cause of Stalin’s death was a hemorrhagic stroke secondary to hypertension [6].

Warfarin hypothesis

For decades, the USSR’s official determination that Stalin’s death was due to natural causes went unquestioned. However, Radzinsky’s 1997 biography of Stalin, as well as Brent and Naumov’s 2003 book detailing the Doctors’ Plot, both raised the intriguing possibility that Stalin’s stroke was instead due to being poisoned by one of his inner circle sometime during the late hours of 28 February or the early morning of 1 March [4,5]. Out of the four men who gathered at the dacha that night, Beria is by far the most obvious suspect [4]. He had recently fallen out of Stalin’s good graces, and the dictator had become “increasingly suspicious” of both him and Khrushchev [17]. Malenkov was also a figure of suspicion for the paranoid dictator - a CIA report after Stalin’s death concluded that, had Stalin lived, it would have only been a matter of time before Stalin “would have set about to destroy him” and his growing power within the Soviet government [18], while Khrushchev had suffered several recent public rebukes in the state press for his agricultural policies [1]. However, out all of these figures, Beria was likely the most vulnerable. Stalin still needed Malenkov for his role in persecuting the Doctors’ Plot [18], and Khrushchev was not as much of a threat as the head of the secret police, which was undergoing a purge at the time [2]. Beria’s star had been publically falling as well. In 1952, he fell from being the fourth Politburo member to enter the opening of the Party Congress to the sixth - a highly visible reprimand that could not have been lost on the USSR’s political elite. Although he was restored to his former position on 21 January 1953 [16], the public embarrassment must have been an unpleasant reminder of the vulnerability of his position. Stalin had a long history of purging even his closest allies, and “after a meeting with Stalin no one ever knew if he would return home alive” [1].

Beria’s behavior was also inordinately suspicious during Stalin’s illness. As previously established, he and Malenkov - unwittingly or not - delayed any medical treatment for Stalin for at least an additional five hours [1]. He was also said to appear “triumphant” during the periods when Stalin was unconscious [17], “glistening with ill-concealed relish” [19] and even bragged to others that “I took him out” [3]. Mere moments after Stalin died, he rushed out of the room, loudly calling for his driver, “the ring of triumph” in his voice [19].

Also suspicious, as Faria pointed out in his 2011 article examining Stalin’s death, are the episodes of hematemesis that Stalin experienced in the latter half of his disease course [6]. As discussed above, physicians at the time were surprised by this development, and even Bulganin, the Minister of Defense, regarded it suspiciously [1]. In the final report of Stalin’s illness to the USSR’s Central Committee, it is notable that any mention of this hematemesis was purged from the record, and only a vague reference to “hemorrhages… in the lining of the stomach and intestine” was made in the autopsy [6].

Faria, as well as Brent and Naumov, suggest that Stalin was poisoned by warfarin (a tasteless and transparent chemical), or a similar anti-coagulant agent, that was slipped into his wine the night of 28 February to 1 March, possibly in a plot by both Beria and Khrushchev [4,6]. The primary reason for this theory, aside from several inconsistencies noted in Khrushchev’s description of the events leading up to Stalin’s death, is the hematemesis that Stalin experienced. Faria concludes that Stalin’s stroke “would not necessarily be associated with gastrointestinal or renal hemorrhaging” [6]. Faria theorizes that the physicians examining Stalin may have even realized that the dictator had been poisoned, but with the “Doctors’ Plot episode… very fresh in their minds” deliberately suppressed the information to avoid “getting their own heads into the repressive Soviet noose” [6]. Even if the Soviet Union had discovered any subsequent evidence of poisoning, it would have been entirely in-character for the government to withhold that information. American intelligence observed that the USSR was extremely careful to avoid any implication of foul play [18], possibly explaining the absence of the hematemesis episode from the government’s official report on Stalin’s death [6]. The continued stability of the Soviet government - and maintaining their own grip on power - was the primary reason that the upper Party cadre delayed any official announcements on Stalin’s illness [1]. To much of Soviet society, “Stalin was portrayed as a god, who of course could do no wrong” [18]. It must have seemed absolutely imperative to silence any rumors that he could have been poisoned by one of his closest lieutenants, in order to prevent any potentially catastrophic social unrest. This fear may have been well-founded; even with his death, Stalin’s cult of personality was so powerful that hundreds were crushed and trampled to death in the vast crowds that attended his funeral [1].

In a 2019 rebuttal to this theory, researchers stated that Beria would likely not have had access to warfarin in the early months of 1953, claiming that warfarin, initially synthesized at the University of Wisconsin, was first marketed in the United States as a rodenticide in 1952, and not approved for human use until 1954, limiting its availability at the time [3]. However, this is incorrect. Warfarin was first marketed as a rodenticide several years earlier in 1948. Even as early as 1951, its use as a potential method of poisoning was documented, when a service member in the US Army attempted suicide with a warfarin overdose [20]. During this timeframe, Soviet intelligence services were arguably still near the very height of their abilities following the “massive espionage campaign” of the 1930s and 1940s that targeted the United States [21]. Obtaining warfarin - a substance that was commercially available five years before Stalin’s death - would have been child’s play compared to penetrating the top secret Manhattan Project [21].

Further supporting the theory of a warfarin poisoning, a supratherapeutic International Normalized Ratio (INR) due to warfarin overdoses does have the potential of causing an acute GI bleed. One study found that 12% of cases of warfarin overdose may manifest with clinical hematemesis, melena, or hematochezia, and noted that “in the pre-endoscopy era, the etiology of bleeding (secondary to warfarin overdose) was often attributed to trivial mucosal lesions” [22] - remarkably similar to the physicians’ conclusions regarding Stalin’s hematemesis [1] as well as his autopsy’s vague references to hemorrhages in the stomach and intestinal lining [6]. In addition to this, warfarin-associated intracerebral hemorrhage is a well-documented medical phenomenon, with approximately 8,000-10,000 cases occurring annually in the United States alone, usually in patients with a mean age in their 70s and a history of elevated blood pressure [23], the same demographic that Stalin was in [2]. Similarly, the risk of intracerebral hemorrhage from warfarin increases as the INR rises [24].

Beria also had a history of poisoning his enemies. In 1936, he invited one of his rivals, Nestor Lakoba, to dinner and poisoned him with an unknown substance. Lakoba died “convulsing” in agony [25]. It would not have been out of character for Beria to have done the same to Stalin, had he been given the opportunity.

Rejecting the poison hypothesis

While Beria likely could have secured access to warfarin and had a history of using poisons to eliminate enemies [25], it appears highly unlikely that Stalin was poisoned by it or a similar anticoagulant. The primary evidence that theorists present for the warfarin-poisoning scenario is Stalin’s episodes of hematemesis [6], which does not hold up to a rigorous review.

The first rebuttal to this theory can be observed in Stalin himself. As his paranoia grew, he became “obsessive about the possibility of being poisoned” [11]. He made his personal chef a general, and had his food “grown and processed at special farms and food factories” [8]. By the end of his life, his paranoia had grown to the point that he regularly “insisted on having his food lab-tested on a retinue of labs and mice that went everywhere with him” [11]. Although there is no reference to this menagerie being present at the dacha the night of 28 February to 1 March, Stalin had a long habit of forcing his guests to drink to excess while he stayed relatively sober [2], and by one account, had only light drinks that night [6]. Even if Khrushchev’s account is correct and Stalin “was a bit tipsy” that night [2], it is difficult to imagine a scenario where Beria or another conspirator could have slipped an anticoagulant into his drink. As Stalin himself stated in 1951, “I trust no one, not even myself” [8]. He never would have given any of his guests - some of whom were under suspicion at the time [2] - such an opportunity.

The second rebuttal is that warfarin is not a particularly effective poison, at least in terms of assassination. By 1951, it was already established that vitamin K could be used to counteract the effects of an acute warfarin overdose. In the case of the US Army inductee mentioned above, his attempted suicide via warfarin overdose was reversed by large doses of vitamin K [20]. With such a widely-available antidote available, it would have been unwise for Beria or any others to use warfarin as a poison. In addition to this, while warfarin overdoses can be associated with acute GI bleeding [22], it is extraordinarily unlikely that Stalin would not have displayed other symptoms of an acute coagulopathy. In 1954, the United States Armed Forces Medical Journal reported a case of a Korean family of 14 that accidentally consumed a mixed corn meal bait laced with warfarin rodenticide. The primary symptoms were ecchymosis and mucosal bleeding [26]. In a 1992 case report, researchers describe a 25-year-old male who attempted suicide by consuming four boxes of rodenticide containing brodifacoum, a vitamin-K antagonist anticoagulant similar to warfarin in its mechanism of action. The patient presented with syncope, hematochezia, hematuria, and epistaxis - he ultimately died of a subarachnoid hemorrhage several weeks later when he returned to the ED after ingesting additional brodifacoum [27]. In addition to this, over the past several decades, there has been an increasing incidence of “superwarfarins” such as brodifacoum contaminating illicit drugs, causing a number of accidental and intentional poisonings. One review article found reports of 16,000 cases of superwarfarins from 2003 to 2006. In many of these cases, patients present with a generalized coagulopathy that often included hematuria, flank pain, easy bruising, hematemesis, and increased bleeding [28]. Aside from his two episodes of hematemesis [7], Stalin did not display any unusual bleeding that is suspicious for an underlying coagulopathy.

Warfarin also has a delayed impact on coagulation and INR, due to the half-life of factors II, VII, and IX in particular. Typically this means that the patient’s INR will not change within 24-48 hours after the administration of warfarin [29]. While this does barely overlap with Stalin’s stroke (assuming he had it after 6:30 PM on 1 March, as discussed above), it would have required Beria to poison the dictator towards the beginning of their gathering. Even if Khrushchev is right in describing Stalin as “tipsy” at the end of the night [2], it would have been significantly more difficult to slip something into his drink unnoticed at an earlier point in the festivities.

It also appears that even if Stalin had been poisoned with warfarin, it would have taken even longer than 24-48 hours for any symptoms to manifest. In the 1954 case of warfarin overdose discussed above, the family continually ate the warfarin-laced bait for several days before manifesting any symptoms, with one of the children consuming the bait for 15 days before she died of a severe “nasal hemorrhage” on the 17th day [26]. Similarly, in the 1992 case report discussed above, the patient who overdosed on brodifacoum was able to delay presenting to the ED until nine days later - even though this compound is 200 times more toxic than warfarin and has a half-life that is 60 times longer [27]. Given this, as well as the massive amounts of anticoagulant consumed in both these cases [26,27], the timeline for Stalin’s alleged poisoning does not line up with his stroke.

Even in the 1951 case, the first recorded poisoning of a human with warfarin, the US Army inductee reported that he consumed warfarin for six consecutive days, consuming approximately a 113-gram carton of warfarin-derived rodenticide during that time. He had no symptoms until two days after his final dose, when he began to experience constipation, epistaxis, back pain, abdominal pain, vomiting, and a petechial rash [30]. Interestingly, he reported that he had learned of the compound from “a recent issue of a popular magazine” [30], supporting our earlier hypothesis that this rodenticide was well-known at the time and that it would have not been outside the realm of possibility for Beria to obtain it. However, this prolonged disease course further emphasizes that Stalin’s symptoms and disease course do not correlate with intentional warfarin poisoning.

The source of the hematemesis

If Stalin’s hematemesis and reported GI hemorrhages were not due to poisoning via warfarin or a similar anticoagulant, then what caused them? A 2011 review article raised the interesting possibility that Stalin may have suffered from stress ulcers or a Mallory-Weiss tear [30]. While Stalin did have a history of stomach pain and intestinal disturbances that increased toward the end of his life [2], possibly consistent with an underlying peptic ulcer or even irritable bowel syndrome [7], his autopsy made no record of underlying ulcers or tears [3]. Other theories include the possibility that the leeches applied to Stalin were responsible for his GI hemorrhage or that he experienced a disseminated intravascular coagulation (DIC). Although these are interesting possibilities, leech bites would not be sufficient to cause a coagulopathy severe enough to induce a GI hemorrhage [3] and the description of Stalin’s symptoms is not consistent with DIC.

The most likely possibility for Stalin’s hematemesis is that it was stress-related, “secondary to the prolonged time interval between the onset of his stroke and his death” [3]. While there is a wealth of research regarding Cushing’s Ulcers - perforation or hemorrhagic erosion of the GI system in patients with significant intracranial diseases - there is little literature regarding the incidence of GI hemorrhages after acute strokes. A 1996 study of 597 stroke patients found that approximately 3% of them experienced GI hemorrhage. In most of these cases, autopsy after death failed to identify any specific source of the hemorrhage [31], consistent with Stalin’s autopsy. Older patients with more severe strokes were more likely to develop GI hemorrhage [31]. The actual percentage of stroke patients with GI hemorrhage may be even higher. Remarkably, a 2002 study of patients with primary ICH found that 30% of patients with ICH experienced a subsequent GI hemorrhage, often manifesting as hematemesis or hematochezia. The researchers theorized that this was associated with stroke severity and the development of septicemia, which could cause “reduction of gastric blood flow leading to mucosal ischemia” [32]. Stalin was recorded to have a white blood cell count of 17,000 in addition to his tachycardia and elevated blood pressure [3], suggesting that he may have developed an underlying septicemia during his treatment. Just as in head trauma patients, the “catastrophic occurrence” of acute ICH may cause GI hemorrhagic ulcers [32]. Ultimately, it appears likely that Stalin’s hematemesis was simply a secondary stress-related reaction.

## Conclusions

Given the evidence currently available to us, it is almost certain that Stalin - a paranoid, inactive, generally unhealthy man in his mid-70s who routinely imprisoned and executed his own physicians, with a longstanding history of chronic untreated hypertension - died exactly the way that the Soviet government declared: from a left MCA hemorrhagic stroke secondary to his hypertension.

It appears highly unlikely that Stalin was poisoned by warfarin or a similar class of anti-coagulant. Although Beria certainly had the motive to assassinate Stalin, the dictator’s death ultimately led to his own several months later. His strange behavior at Stalin’s bedside can either be attributed to Khrushchev deliberately altering the facts for his own political advantage, or due to genuine relief that Stalin was dying. In addition to this, the description we have of Stalin’s illness does not match the appearance or timeline of patients that experience severe warfarin or warfarin-related overdoses. His hematemesis does not appear to be associated with any other generalized coagulopathy, and was likely stress-related. While Beria and Malenkov may have deliberately stalled Stalin’s care during the morning of 2 March, it appears highly unlikely that either of them played a role in deliberately poisoning the dictator. Ultimately, it appears that Joseph Broz Tito, the dictator of the communist regime of Yugoslavia, was correct when he pronounced upon Stalin’s death: “Nature is the ally of justice.”
